# Supplementary material for: Diversity of Pectobacteriaceae Species in Potato Growing Regions in Northern Morocco
Source: Microorganisms. 2020 Jun 13;8(6):895. doi: 10.3390/microorganisms8060895 (PMC7356628; doi:10.3390/microorganisms8060895)

Fig S1. Phylogenetic analysis of the *gapA* barcode.

This figure shows a relation tree of *gapA* barcode of Moroccan populations and other *Pectobacterium* and *Dickeya* strains (sequences were collected from NCBI database). The alignment was generated using ClustalW; the evolutionary history was inferred using the Neighbor-Joining method and the evolutionary distances were computed using the Maximum Composite Likelihood method. Phylogenetic analyses were conducted using MEGA7 software.

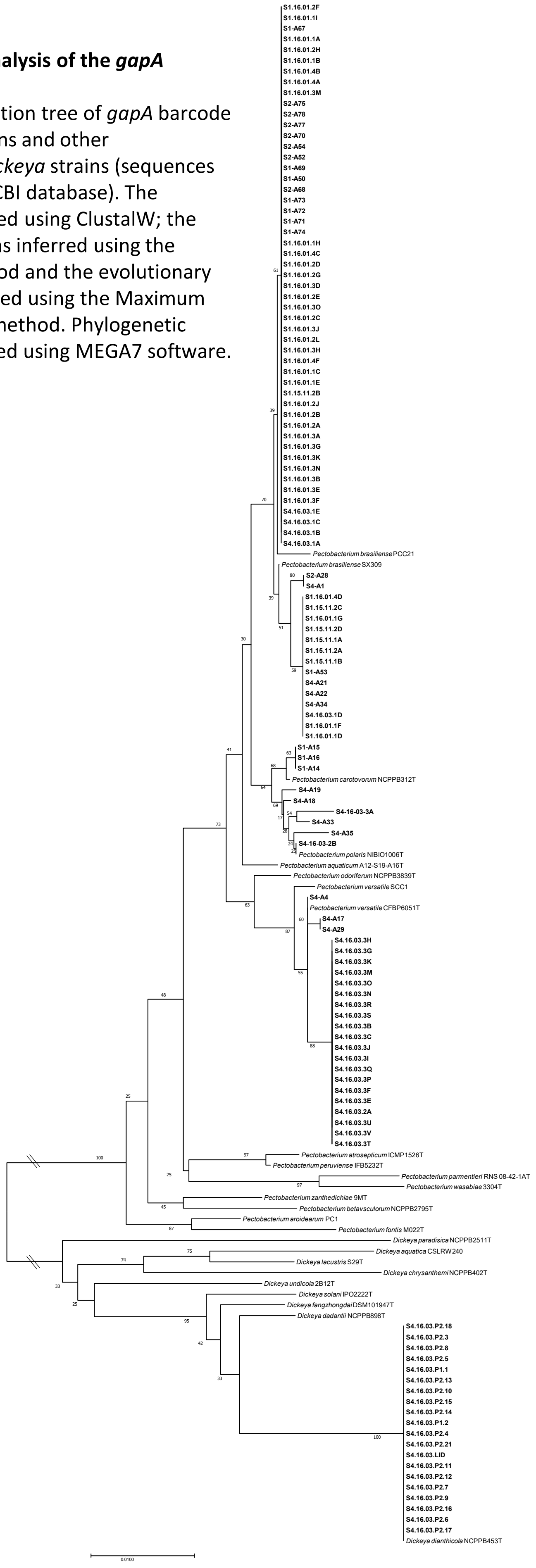

Supplement: Supplementary file 1 [file microorganisms-08-00895-s001.zip › microorganisms-802802-si-proof-done/figS1.pdf]
